# Heme as activator and target for artemisinin: Towards multiple pharmacological bioactivity

**DOI:** 10.1016/j.bbrep.2025.102398

**Published:** 2025-12-09

**Authors:** Pan Zhu, Xinyi Sun, Yuting Fang, Yufei Li, Liang Guo

**Affiliations:** a School of Life Sciences and Health Engineering, Jiangnan University, Wuxi, 214122, China; b School of Life Science, Shanxi Normal University, Taiyuan, 2030031, China

**Keywords:** Artemisinin, Heme, Malaria, Cancer, Mechanism

## Abstract

Artemisinin, a sesquiterpene trioxane derived from the plant *Artemisia annua*, possesses a distinctive peroxide bridge structure that endows it with remarkable biological activity, primarily through its interaction with heme. Given artemisinin's well-established clinical safety profile, researchers are increasingly investigating its potential applications in antifungal, anticancer, and antiviral treatments. This review employs heme as a foundational element to dissect the mechanisms underlying the diverse pharmacological actions of artemisinin, thereby expanding its application prospects. It also offers valuable insights for the development of novel pharmaceuticals and innovative therapeutic strategies targeting various human diseases.

## Graphical abstract

**Figure:**
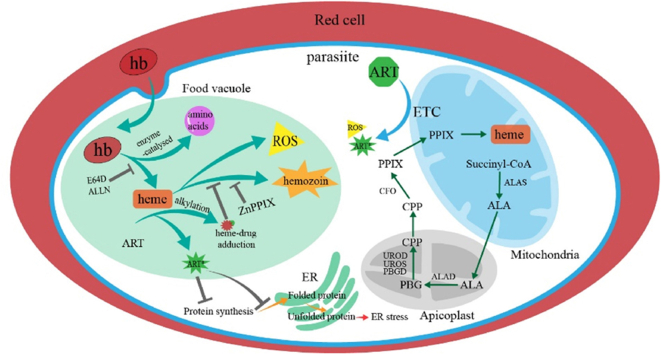

## 1 Introduction

Malaria, an infectious disease instigated by the *Plasmodium* parasite, is primarily transmitted through the bites of *Anopheles* mosquitoes [1]. As a pressing global public health concern, malaria continues to pose a significant threat to human health, necessitating ongoing vigilance and concerted efforts for its prevention and control. As reported by the World Health Organization (WHO), in 2022, there were approximately 249 million cases of malaria [2]. In recent years, the mortality rate has exhibited a gradual decline, a trend attributed to the mosquito control measures instituted at the turn of this century and the advent of more effective pharmacological therapies for the treatment and prevention of malaria [3]. While several countries in Asia and Latin America have successfully ceased malaria transmission, Africa continues to be a region with a significant burden of malaria cases and a major contributor to related fatalities. A principal factor in the persistence of malaria is the escalating resistance of *Plasmodium* to most existing antimalarial medications, particularly chloroquine and sulfadoxine-pyrimethamine [4,5]. The emergence of artemisinin and the formulation of artemisinin-based combination therapies (ACTs) have instilled renewed hope for malaria control. Furthermore, there is an increasing body of evidence suggesting that artemisinin may also be efficacious in treating various other diseases, displaying potential antiviral, antifungal, anticancer properties, and treating polycystic ovarian syndrome, obesity, and diabetes [[6], [7], [8], [9], [10]].

Ancient Chinese medical manuscripts, notably Ge Hong's "Zhou Hou Bei Ji Fang" from the Eastern Jin Dynasty and Li Shizhen's "Ben Cao Gang Mu" from the Ming Dynasty, both document the utilization of artemisinin in the treatment of malaria [17]. In light of these findings, China initiated a drug discovery program in 1967, during which various herbal formulations were evaluated, leading to the revelation in 1971 that extracts from *Artemisia annua* exhibited anti-malarial activity in murine models [18]. In the subsequent years, the active component of this extract was isolated, and several artemisinin analogs were identified, notably artesunate, artemether, and dihydroartemisinin. Each of these compounds features a peroxide bridge, which is essential for their anti-malarial efficacy [19]. The distinct profiles of various derivatives may differ. For example, artesunate, characterized by its excellent water solubility and high bioavailability, proves particularly advantageous for the emergency treatment of severe malaria [20]. Nevertheless, artemisinin-based therapeutics are distinguished by their rapid action, remarkable potency, low toxicity, and abbreviated half-life, rendering combination therapy with long-acting anti-malarial agents an optimal and endorsed strategy [21]. Beyond their pharmacological attributes, elucidating the mechanism of action of artemisinin is vital for the effective treatment of malaria and the refinement of therapeutic protocols. However, the current comprehension of the anti-malarial mechanisms of artemisinin remains inadequate,and this review chiefly explores the mechanism associated with heme.

Heme (Fe^2+^ protoporphyrin IX) is a ubiquitous and vital tetrapyrrole molecule that fulfills a myriad of essential physiological roles, including oxygen transport, electron transfer, and transcriptional regulation [22]. Recognized for its high redox activity, heme is increasingly acknowledged as an activator and target of antimalarial endoperoxides [23]. The peroxide moiety within artemisinin engages in a reaction with planar achiral ferrous (II) heme, and the reductive activation of the peroxide functional group produces a transient alkoxy radical that swiftly rearranges into a carbon-centered primary radical. This radical alkylates heme through an intramolecular mechanism, resulting in the formation of covalent heme-drug adducts. The accumulation of non-polymerizable, redox-active heme derivatives consequent to heme alkylation is regarded as toxic to the parasite. Evidence of heme alkylation by artemisinin in malaria-infected murine models has been established, signifying that heme functions as both an initiator and a target for artemisinin [24].

This review investigates the advancements in the application of artemisinin and its derivatives across diverse biomedical domains, with particular emphasis on their mechanisms in relation to heme. It aims to illuminate the research progress of artemisinin in the realms of anti-malarial, anti-parasitic (beyond malaria), antifungal, anti-cancer, and antiviral interventions. Furthermore, it offers fresh perspectives for the development of new pharmaceuticals and innovative therapeutic strategies.

## 2 The role of heme in the antimalarial effects of artemisinin

Since 1972, artemisinin has been extensively employed in the treatment of patients afflicted with malaria, and it continues to serve as a lifesaving intervention in sub-Saharan Africa [5]. However, the extended use of artemisinin has led to the emergence of resistance in *Plasmodium* and a reduced sensitivity to artemisinin-based therapies in this direction. The investigation of artemisinin resistance is intrinsically linked to the mechanisms underlying antimalarial action, and in recent years, researchers have been actively engaged in thorough studies. Among their findings, it has been determined that artemisinin resistance correlates with mutations in the Kelch protein, which diminishes the endocytosis of hemoglobin and consequently hampers the activation of artemisinin, resulting in resistance [25,26]. In addition, apart from genetic mutations, alternative adaptive mechanisms can contribute to the development of drug resistance. Research indicates that alterations in the expression of heme oxygenase 1 (HO-1) can impact cellular drug sensitivity. Specifically, a reduction in HO-1 levels has been found to reverse resistance to osimertinib in cells [27]. Experiments have shown that the addition of hemozoin-inhibiting drug triarylimidazole14c can lead to a significant increase in heme levels and a corresponding decrease in artemisinin activity. The introduction of heme oxygenase into malarial parasites counteracted the increase of heme and the effect of triarylimidazole14c on artemisinin [28]. These findings suggest that HO can detoxify excessive heme, potentially influencing the drug sensitivity of artemisinin. Through its catabolic activity, HO-1 can prevent heme-induced inflammation and enhance disease tolerance by combining its immune regulation and cytoprotective ability at the erythrocyte stage [29]. In the liver stage, HO-1 protects the infected hepatocytes from death, thereby reducing resistance to infection and leading to a significant increase in the number of parasites progressing to the red cell stage of the parasite life cycle [30].Additionally, recent findings propose that artemisinin heme adducts play a crucial role as metabolites in the emergence of drug resistance in *P. yoelii* [31]. Earlier studies have postulated the antimalarial activation of artemisinin by heme [32]. The current association between heme and artemisinin-based antimalarials unveils further potential avenues for exploration.

### 2.1 Heme pathways in malaria

*P. falciparum* acquires nutrients from host red blood cells through the degradation of hemoglobin, a process that releases substantial amounts of free heme within the parasite. This accumulation can induce lipid peroxidation and ultimately result in cell death. Consequently, *Plasmodium* has evolved a distinctive mechanism for heme detoxification and employs multiple strategies to regulate heme levels.

*Plasmodium* possesses a conserved heme biosynthesis pathway [33]. Heme is synthesized through a sequence of eight enzymatic reactions. In *Plasmodium*, the initial step involves the synthesis of δ-aminolevulinic acid (ALA) from glycine and succinyl-CoA, while the final step occurs in the mitochondria, where protoporphyrin IX combines with an iron ion to produce heme. This pathway has been identified as an essential mechanism in the parasite [34]. Succinyl acetone, an inhibitor of the heme synthesis pathway, has been shown to impede parasite growth at millimolar concentrations. While, ferrochelatase as a crucial enzyme in the heme synthesis pathway, has been knocked out in both rodent and human malaria models [35,36], with no significant changes observed in the growth of the ferrochelatase knockout strain compared to the wild-type strain. Furthermore, the use of ^13^C-labeled ALA demonstrated that low concentrations of succinyl acetone could inhibit heme synthesis but did not affect parasite growth [37]. This suggests that high concentrations of succinyl acetone inhibit parasite growth through off-target effects rather than via the heme synthesis pathway. These findings strongly indicate that the heme synthesis pathway may not be essential during the blood stage of the parasite's life cycle.

*P*. *falciparum* infects red blood cells and absorbs hemoglobin from the host through endocytosis, degrading 60–80 % of the hemoglobin, this process serves as a vital source of amino acids for the parasite [38]. However, the degradation of hemoglobin results in the release of substantial amounts of heme, which in turn generates reactive oxygen species (ROS)—these are detrimental to both the parasite and the host. In response, the parasite has evolved a range of defensive mechanisms to mitigate the harmful effects of elevated heme levels, one of which is the polymerization of heme into hemozoin [39]. The process of hemoglobin degradation and hemozoin formation necessitates a diverse array of catalytic proteases that facilitate the breakdown of hemoglobin into amino acids and heme. Among these enzymes are the cysteine proteases falcipain (specifically falcipain 2A and falcipain 2B), the aspartic acid proteases plasmepsin II, III, and IV, and the metalloproteinase falcilysin, as well as heme detoxification enzymes and aminopeptidases [40,41].

### 2.2 Heme-activated promiscuous mechanism of artemisinin in malaria

Artemisinin features a peroxide bridge that, upon activation, is disrupted and recombined to yield a free radical. This free radical subsequently alkylates malarial proteins, rendering them inactive. Activated artemisinin inhibited protein synthesis and obstructed the proper folding of newly synthesized proteins. The accumulation of improperly folded proteins in the endoplasmic reticulum induces ER stress, which may ultimately culminate in cell death [42]. Furthermore, it alkylates heme to generate heme-drug adducts, which are cumulatively toxic to *Plasmodium* [24]. Heme-artemisinin adducts exert their action during the trophozoite stage of the parasite's life cycle through a secondary mechanism that inhibits the formation of hemozoin, thereby thwarting the parasite's ability to convert toxic heme into non-toxic hemozoin. This disruption leads to heme accumulation within the parasite, consequently elevating its toxicity [43]. These findings indicate a correlation between heme and artemisinin.

Thus, it appears that elevated heme levels potentially augment the activation of artemisinin, thereby enhancing its antimalarial efficacy. Utilizing a chemical proteomics approach, a research team examined artemisinin through its active probe (AP1) and demonstrated that artemisinin is particularly effective during the trophozoite and schizont stages of *Plasmodium*, which release markedly high levels of heme through the degradation of hemoglobin [44]. Similarly, analyses of blood samples from patients with normal and severe malaria revealed that heme levels were elevated beyond normal ranges. And heme significantly enhanced the antimalarial activity of artemisinin while reducing the half-maximal inhibitory concentration (IC_50_) of the drug at this pathological concentration [45]. Elevated heme levels may provide a potentiating effect on the antimalarial properties of artemisinin. Given the connection between the antimalarial mechanism of artemisinin and heme, it is reasonable to conjecture that high heme concentrations may further amplify the effects of artemisinin.

### 2.3 The arguments for considering heme as an activator of artemisinin

However, the role of heme as an activator of artemisinin remains a subject of some controversy. An experiment revealed that the process of hemoglobin degradation is not necessarily correlated with artemisinin sensitivity during the trophoblastic phase. This was demonstrated using a parasite mutant deficient in initiating hemoglobin hydrolysis, which exhibited an unexpected sensitivity to artesunate despite its reduced heme content [46]. Moreover, the administration of the hemoglobin degradation pathway inhibitor Pestatin A and RO 40–4388 did not yield any significant impact on artemisinin sensitivity. This raises doubts about whether the production of heme influences artemisinin sensitivity [47]. Furthermore, a recent study revealed that elevating *Plasmodium*-free heme levels through the hemozoin formation inhibitor triarylimidazole 14c (hereafter referred to as 14c) resulted in a significant increase in the EC_50_ of artemisinin. The extracellular addition of heme analogs similarly antagonized the effects of artemisinin [28]. This indicates that elevated heme levels may exert an antagonistic effect on artemisinin, in stark contrast to the previous enhancement of artemisinin's efficacy attributed to high heme concentrations.

In recent years, a significant debate has emerged regarding the impact of heme on the activity of artemisinin. On the one hand, heme enhances the activity of artemisinin. Heme, a by-product of hemoglobin digestion of by *Plasmodium*, plays a crucial role in the activation of artemisinin. Heme can form active complexes with artemisinin, thus enhancing its antimalarial efficacy of artemisinin. Evidence indicates a correlation between elevated heme level and increased artemisinin effectiveness, especially during advanced stages of hemoglobin degradation in the life cycle of *Plasmodium*, further reinforcing this perspective. On the contrary, heme antagonizes the activity of artemisinin. Heme itself does not have antimalarial properties, but may reduce the effectiveness of artemisinin. The observation that inhibiting heme degradation unexpectedly enhances the activity of artemisinin provides support for this perspective. These findings indicate that the relationship between heme and artemisinin is intricate and influenced by many factors, such as experimental conditions, *Plasmodium* species, artemisinin concentration and the nature of the interaction between heme and artemisinin. Therefore, it is inaccurate to broadly assert that heme consistently enhances or antagonizes the activity of artemisinin. The effect of heme on artemisinin should be evaluated under specific experimental conditions and the background of the research subject. Future investigations should delve into the mechanism underlying the interaction between heme and artemisinin, explore how fluctuations in heme concentration in the physiological or pathological range to regulate the efficacy of artemisinin, investigate whether external molecules in the host or parasite environment influence the heme-artemisinin interaction, and ultimately optimize artemisinin-based antimalarial therapies.

### 2.4 Heme homeostasis: therapeutic implications and off-target toxicity risks

The safety of artemisinin-based antimalarial drugs is a critical consideration due to their heme-activation-dependent mechanism of action, which can disrupt systemic heme homeostasis, potentially leading to unintended toxicity. Treatment with artesunate has been associated with delayed hemolytic anemia, a phenomenon linked to heme levels. In cases of pulmonary malaria, heme derivatives affect the generation of inflammatory mediators and the infiltration of inflammatory cells into tissues [48,49]. Patients deficient in glucose-6-phosphate dehydrogenase (G6PD) characterized by reduced erythrocyte antioxidant capacity are particularly vulnerable to artemisinin-induced oxidative stress, which can precipitate acute hemolytic anemia, heightening treatment risk [[50], [51], [52]]. Therefore, caution is warranted in utilizing artemisinin derivatives in clinical settings, necessitating G6PD deficiency screening in patients and vigilant monitoring of post-treatment hemolytic parameters.

### 2.5 Summary

Heme plays a significant role in the antimalarial action of artemisinin (seeFig. 1). Prior experiments have demonstrated that heme activates artemisinin and that the sensitivity to artemisinin is intricately connected to the metabolic processes of hemoglobin. However, the precise mechanisms involved remain somewhat ambiguous, and certain studies yield conflicting results, highlighting critical issues that require urgent attention in this field.

**Fig. 1:**
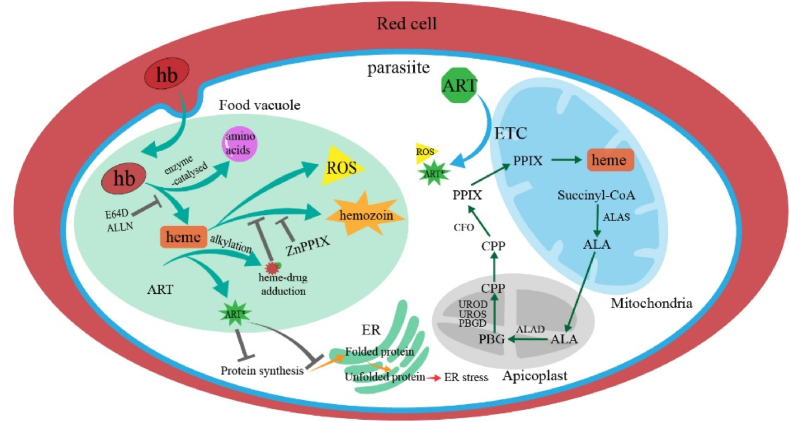
Heme related pathways in malaria which participate in artemisinin antimalaria action.

## 3 The role of heme in the antifungal effects of artemisinin

In recent years, owing to sustained and thorough investigations into artemisinin-based compounds, scientists have unveiled that artemisinin and its derivatives exhibit not only antimalarial properties but also antifungal and other effects. In vitro studies have illustrated that extracts of *A*. *annua* exhibit antimicrobial activity against a broad spectrum of pathogenic microorganisms, including *Bacillus subtilis*, *Escherichia coli*, *Candida krusei*, and *Staphylococcus aureus* [53,54].

### 3.1 The antifungal mechanism of artemisinin

The antifungal activity and mechanisms of artemisinin have garnered significant attention from researchers. Galal AM et al. determined the inhibitory effects of artemisinin and its derivatives against *C*. *albicans* and *Cryptococcus neoformans* [55], and Elfawal MA et al. discovered that artemisinin exerts an inhibitory effect on *Candida* at high concentrations [56]. It has been demonstrated that silver nanoparticles synthesized from extracts of *Artemisia* leaves could significantly reduce the ergosterol content of *Candida*, compromise the integrity of *Candida* cell membranes, and display remarkable antifungal activity against prevalent clinical species of *Candida* [57]. The investigations conducted by De Cremer K et al. and Das S et al. revealed the accumulation of ROS during the action of artemisinin and its analogs on *C. albicans*, indicating that the generation of ROS is a key mechanism through which artemisinin manifests its antifungal efficacy [58,59]. Screening for compounds that disrupt fungal metal homeostasis in *C*. *albicans* revealed that artemisinin was capable of diminishing total intracellular iron and unstable zinc levels. Consequently, it was postulated that artemisinin might influence zinc-iron homeostasis, thereby facilitating fungal inhibition [60]. There is a noteworthy observation that various plant extracts, including artemisinin, can effectively inhibit *C*. *glabrata*. Moreover, investigations utilizing the *Saccharomyces cerevisiae* model have unveiled an increased sensitivity in the knockout strains of PDR5, SNQ2, and YOR1 [61]. In *S. cerevisiae*, artemisinin influenced mitochondrial function, leading to a swift and substantial production of ROS and the depolarization of the mitochondrial membrane potential, thus manifesting its fungicidal effects [[62], [63], [64]]. Furthermore, it has been demonstrated that artemisinin could enhance the activity of amphotericin B against *C. albicans* by activating the ergosterol synthesis pathway [65], while artemether can amplify the efficacy of fluconazole by disrupting the function of the efflux pump PDR5 [66]. These findings provide a framework for further studies on the antifungal mechanism of artemisinin. There were several findings utilizing yeast models to investigate the mechanisms of artemisinin. The heme pathway and the mitochondrial pathway discussed in this article are two prominent areas of research. It is recognized that cytochrome *c* is an integral protein situated within the inner mitochondrial membrane, covalently bonded to heme and apolipoprotein [67]. This leads to the speculation that heme may indeed have a role in the mitochondrial pathway.

### 3.2 Heme-mediated effect of artemisinin on fungi

It was hypothesized that heme plays a crucial role in the activation of artemisinin [68]. Jasmin et al. demonstrated that the biosynthesis of heme is a prerequisite for the cytotoxic effects of artemisinin, as evidenced by screenings conducted with yeast and haploid stem cells [11]. Sun Chen et al. found that sufficient levels of heme may induce cytotoxicity by reacting with artemisinin to generate ROS [63]. In fermentation media, elevated concentrations of dihydroartemisinin inhibit yeast growth, a phenomenon that is dependent on heme, suggesting that heme mediates the anti-yeast activity of dihydroartemisinin [69]. Furthermore, Bairwa et al. engineered a chromosome-encoded heme sensor for *S. cerevisiae* employing fluorescence microscopy among various other techniques. This sensor facilitates a deeper exploration of the intricate relationship between artemisinin activity and heme [70].

### 3.3 Mitochondria-mediated effect of artemisinin on fungi

By utilizing the yeast *S. cerevisiae* as a model organism, Li W et al. illustrated that artemisinin induces depolarization of mitochondrial membranes, whereby enhanced electron transport activity renders cells increasingly susceptible to artemisinin [62]. Laleve A et al. discovered that a fluorescent dihydroartemisinin probe could be directed towards yeast mitochondrial cytochrome *c*, thereby demonstrating that reduced mitochondrial c-type cytochromes serve as targets that facilitate the activation of artemisinin within yeast cells [71]. While, a reduction in heme levels in non-fermented media may lead to heightened susceptibility to artemisinin [69]. In other words, the regulation of heme levels under conditions necessitating mitochondrial respiratory function can modify susceptibility to artemisinins. This suggests the possibility of a competitive interplay between heme and mitochondrial pathways when artemisinin operates in non-fermented media [72]. In addition, in the *Galleria* infection model, treatment with artemisinin resulted in a significant increase in ROS, the release of cytochrome *c* from mitochondria and its subsequent accumulation in the cytoplasm, along with the significant loss of mitochondrial membrane potential. Artemisinin may interact with the Fe center of cytochrome *c* to form a free radical complex, ART-cyt *c*, which facilitates the translocation of cytochrome *c* across the mitochondrial membrane [73]. The uncoupled cytochrome *c* is then released from the dysfunctional mitochondrial membrane into the cytoplasm, where it activates a caspase-3-like protease activity dependent apoptosis pathway, thereby exerting an antibacterial effect [74].

### 3.4 Summary

In addition to the well-established antimalarial efficacy of artemisinin, its potential for antifungal applications is especially remarkable (seeFig. 2). The pivotal role of heme in the mechanism of action of artemisinin opens fruitful avenues for the development of novel antifungal agents. These findings not only enhance our understanding of the mechanisms underlying artemisinin's effects but also chart a new course for the development and clinical application of antifungal therapies. With continued research, artemisinin and its derivatives are anticipated to emerge as effective options for the treatment of fungal infections.

**Fig. 2:**
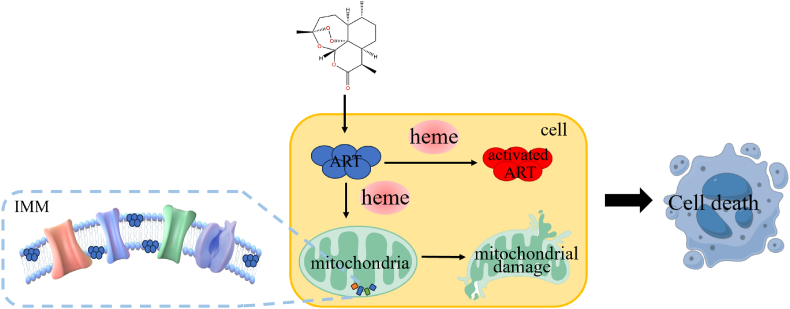
Heme and mitochondria mediated artemisinin antifungal activity.

## 4 The role of heme in the anticancer effects of artemisinin

In recent years, there has emerged an increasing fascination with the repurposing of artemisinin and its derivatives for the treatment of non-parasitic ailments, notably cancer. This burgeoning interest is rooted in their established chemical and pharmacokinetic characteristics. Investigations have demonstrated that it is effective against various types of cancer, including lung, breast, liver, colorectal, ovarian, esophageal, and gastric cancers [75]. The potential of artemisinin as an anticancer agent was initially reported in 1993, and since that time, a multitude of studies have evidenced its conceivable anticancer efficacy [[76], [77], [78], [79], [80], [81]].

### 4.1 Heme-activated anticancer mechanism of artemisinin

Previous meta-analyses on heme iron and cancer have indicated that a significant positive correlation between heme iron intake and the incidence of colorectal cancer, colon cancer [82] and breast cancer [83]. Although no association has been found between heme intake and lung cancer risk [84], high heme intake is associated with an increased risk of various cancers [85].

The endoperoxide bridge of artemisinin can engage with Fe(II) or heme, thereby catalyzing the transfer of oxygen atoms from the moiety, ultimately leading to the generation of ROS and carbon-centered free radicals [86]. The levels of heme in tumor cells are higher compared to those in normal cells, making tumor cells more susceptible to artemisinin's effects [87]. In normal cells, heme levels are regulated and maintained at a relatively low concentration, which may contribute to the specific cytotoxicity of artemisinin observed in cancer cells [12]. Research has demonstrated that enhancing intracellular heme synthesis could amplify the cytotoxicity of dihydroartemisinin to cancer cells, whereas the attenuation of heme synthesis through the introduction of succinyl acetone reduced this toxicity [22].To explore the role of iron and heme in the activation of artemisinin, researchers introduced holotransferrin as an intracellular source of Fe (II), which resulted in the elicitation of artemisinin-induced cytotoxicity. Upon the addition of succinylacetone, a known heme inhibitor, the toxicity instigated by holotransferrin was diminished, leading to the conclusion that iron contributes to cellular toxicity through its involvement in heme synthesis [88]. This suggests that active heme serves as a principal activator of artemisinin within cancer cells [68]. Its interaction with artemisinin may prove to be more efficacious than with other iron-containing entities, including Fe (II), various inorganic iron compounds, and hemoglobin. Furthermore, the designation "active heme" in this context typically pertains to free heme, owing to its markedly superior capacity to activate artemisinin compared to heme-protein complexes [88]. The endoperoxide bridge may interact with electrons on heme, thereby activating artemisinin and prompting the cleavage of the endoperoxide bridge, resulting in the generation of alkoxy radicals capable of alkylating proteins and macromolecules within the cell, thus altering their structure and inducing DNA breaks in tumor cells [44,89]. Artemisinin precipitates a decline in mitochondrial membrane potential and facilitates the production of ROS [90]. Furthermore, the heme-catalyzed endoperoxide bridge of artemisinin also generates ROS, and the accumulation of excessive ROS can activate caspases, thereby enhancing apoptotic signals, inflicting damage on organelles and intracellular molecules, and ultimately culminating in cell death [91,92].

In a study of esophageal squamous cell carcinoma, treatment with 5-ALA led to the inhibition of glutathione peroxidase 4 (GPX4), a crucial antioxidant enzyme, and upregulation of HO-1 expression in the cells [93]. This dual mechanism facilitated the acceleration of ferroptosis in the tumor cells.

Artesunate induces iron-dependent, ROS-mediated, and caspase-independent cell death in cancer cells, promoting ferroptotic cell demise. Furthermore, during prolonged oxidative stress, Keap1, a suppressor of Nrf2, inhibits the Keap1-Nrf2-antioxidant response element (ARE) pathway [94]. This inhibition enhances cancer cell susceptibility to therapies targeting ferroptosis but may also diminish their sensitivity to artesunate [95]. This pathway plays a pivotal role in driving ferroptosis in hepatocellular carcinoma cells [96]. As a specific inducer of ferroptosis, artesunate holds significant potential for broader application in cancer therapy and surmounting drug resistance in cancer cells [97].

The metabolism of heme by HO-1 results in the formation of bilirubin, which has been identified in a meta-analysis as a potential biomarker for inflammation and oxidative stress in rheumatic diseases [98]. Furthermore, the endogenous antioxidant properties of bilirubin may also contribute to the preliminary diagnosis of tumor cells and the anticancer activity of artemisinin.

### 4.2 The synergistic role of heme with artemisinin in anticancer therapy

In a Phase I trial investigating the efficacy of artesunate pessaries in treating Grade 2 or 3 cervical intraepithelial neoplasia (CIN2/3), an increase in the area under the plasma concentration-time curve (AUC) of artesunate (AS) was observed. Following five consecutive days of administration, both the time to reach maximum concentration (Tmax) and the half-life of artesunate were prolonged [99]. In a separate clinical investigation (NCT02354534) involving intravaginal artesunate suppositories for the treatment of HPV-positive high-grade cervical intraepithelial neoplasia (CIN2/3) in women aged 18 to 65, without surgical intervention, 50 % of participants tested negative for the HPV genotype. Additionally, 62.5 % experienced histological regression from CIN2/3 to CIN1 or lower after two five-day cycles of 200 mg artesunate suppositories. While no severe adverse events were documented, some participants reported adverse effects such as tinnitus, abdominal pain, and nausea.

In experiments involving colorectal cancer, it was observed that heme levels in cancerous cells exceeded those in non-cancerous cells, and ALA synthase exhibited heightened expression in the cancerous cells [12]. Most artemisinin combination therapies operate by enhancing heme synthesis, thus elevating ROS levels to trigger cell death. The chemotherapeutic agent gemcitabine is partially metabolized by cytidine deaminase (CDA) within the body, consequently diminishing its therapeutic efficacy. When dihydroartemisinin is administered in conjunction with gemcitabine, ROS produced from the activation of peroxide in the presence of heme promote the expression of heme oxygenase-1 while concurrently inhibiting the expression of CDA [100], thereby amplifying the anticancer efficacy of gemcitabine [101]. Furthermore, it demonstrates reduced hematologic toxicity compared to GEM monotherapy. Moreover, ALA, recognized as a clinically utilized precursor for heme synthesis, is employed to enhance heme levels [102]. The therapeutic effect of the artemisinin and ALA combination therapy was markedly more potent than that of artemisinin administered alone [12]. In two highly aggressive brain tumor models, the combination of 5-ALA and dihydroartemisinin induced tumor cell death by elevating levels of ROS and inducing DNA damage. In mice treated with 5-ALA and artesunate, the activity of the heme biosynthesis pathway in patient-derived tumor allograft glioblastoma cells was significantly greater than that observed in normal cells. Furthermore, the combined treatment with 5-ALA and DHA led to the specific ablation of GFP-positive regions in glioblastoma, which was not achieved with individual agents alone [11]. This study provide evidence that 5-ALA and artesunate exhibit robust synergistic activity in human-relevant glioblastoma models in vivo [11]. Moreover, given the established safety profiles of both artemisinin and 5-ALA in relevant patient populations, their combined application in brain tumors appears relatively safe [11]. Similarly, the conjunction of histone deacetylase inhibitors and artesunate has the potential to elevate the expression of ALA synthase, thereby facilitating heme production and enhancing the cytotoxic effects of artesunate on tumor cells [103]. Artemisinin combination therapies have underscored their efficacy in the treatment of colorectal cancer, glioblastoma, and cervical cancer, among other malignancies [[11], [12], [13]].

### 4.3 The synergistic role of heme with artemisinin in anticancer photodynamic therapy

Photodynamic therapy (PDT) serves as an effective modality for cancer treatment and has garnered notable success in the management of esophageal cancer [104,105]. Heme-activated artemisinin directly generated ROS that augment the efficacy of PDT [106,107]. The combination of artemisinin and its derivatives with PDT has emerged as a novel strategy for cancer treatment. It is well established that artemisinin can facilitate the accumulation of ROS within cells via heme, thereby mediating caspase-dependent apoptosis to eliminate breast cancer cells [108]. Given that the addition of 5-ALA could elevate intracellular heme levels and amplify the activation of artemisinin in cancer cells, the combination therapy of artemisinin and 5-ALA may prove to be more efficacious than artemisinin administered alone [109]. Subsequent experiments revealed that, in contrast to artemisinin, artemether may more significantly augment 5-ALA-PDT-induced apoptosis in murine breast tumor cells [110]. In a recent study, a pH-responsive lipidosome was formulated to co-deliver ALA and artemisinin. This lipidosome exhibited notable stability in the circulatory system. Upon administration in a murine breast cancer model, substantial decrease in tumor size was observed within 48 h. Moreover, compared with the control group, both the infiltration rate of cytotoxic T lymphocytes and the expression levels of cytokines (such as TNF-α and IFN-γ) were increased. These results collectively demonstrate the effective anti-tumor efficacy of this liposome in vivo [110]. Additionally, the formulation displayed exceptional biocompatibility and enhanced bioavailability [110]. This further underscores the feasibility and efficacy of developing artemisinin-based combination therapies that target the heme pathway for the treatment of cancer.

### 4.4 Heme-mediated systemic toxicity and therapeutic efficacy

The disruption of heme homeostasis is a common feature of tumor cells [111]. Under normal physiological conditions, the liver synthesizes heme while maintaining precise biological control to avoid toxicity. In contrast, tumor cells exhibit a dysregulated process of heme biosynthesis, where the upregulation of heme biosynthesis genes leads to the accumulation of intermediate porphyrins, a feature absent in healthy tissues [112].

Factors influencing the tumor immune microenvironment (TIME) include the generation and response to damage-associated molecular patterns (DAMPs). Labile heme, released from lysed red blood cells or necrotic cells, functions as a DAMP that promotes inflammation and cancer progression through interactions with Toll-like receptors (TLRs) [113]. Although normal free heme is not cytotoxic, it can mediate programmed cell death in non-hematopoietic cells, particularly in response to pro-inflammatory agonists such as TNF [114]. Numerous genes associated with a pro-transformation phenotype have been shown to be regulated by heme, linking them to immunomodulatory functions that also affect the TIME [115]. Excess heme can diminish the function of the tumor suppressor protein p53 by directly binding to it, thereby disrupting the p53-DNA interaction. Notably, heme exerts opposing effects on p53 expression in tumor cells compared to normal cells, and its impact on tumor cells is heterogeneous. For instance, in colon cancer cells, high concentrations of heme lead to decreased p53 expression, thereby promoting tumorigenesis. Conversely, in normal colon epithelial cells, exposure to high heme concentrations upregulates p53 expression, thereby inhibiting tumor formation. Thus, heme-dependent p53 expression appears to differ between non-neoplastic and cancer cells [116].

In most experimental settings, the HO-1 inhibitor Zinc Protoporphyrin IX (ZnPPIX) has been demonstrated to increase the susceptibility of cancer cells to apoptosis and enhance their sensitivity to cancer therapies. Furthermore, when combined with DHA, ZnPPIX exacerbates the degree of DHA-induced ferroptosis [13]. In both B16 melanoma and 4T1 breast cancer cell lines, ZnPPIX effectively increases intracellular levels of labile heme, thereby augmenting the capacity of DHA to generate ROS and inhibit tumor growth [117].

### 4.5 Summary

**Fig. 3:**
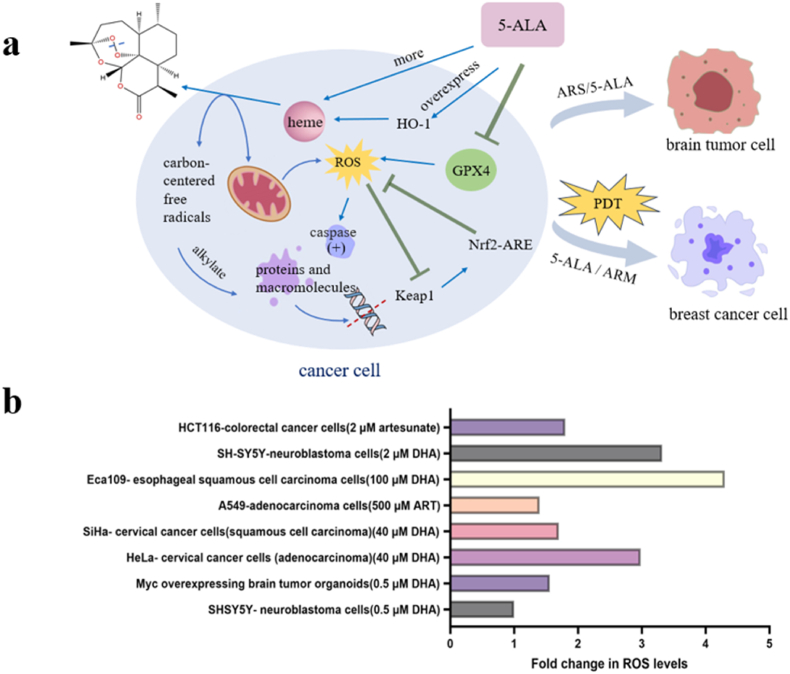
Anticancer mechanism of artemisinin. (a) Heme as an activator for artemisinin action in cancer therapy. (b) ROS levels in tumor cells following treatment with artemisinins. Data source [[11], [12], [13], [14], [15], [16]].

In summary, heme serves as the primary catalyst for the cleavage of the endoperoxide bridge in artemisinin, and the resultant elevation of ROS levels and DNA damage ensuing from this cleavage, have been corroborated in a variety of tumor cells [44,108,118,119] (see Fig. 3). Artesunate, as a specific activator of ferroptosis, induces cell death through ferroptosis. Many studies have utilized heme as a primary activator, employing drugs such as the heme synthesis precursor ALA to increase intracellular heme levels. This leads to the inhibition of GPX4 and high expression of HO-1 and have uncovered additional combinable therapies while probing into their mechanisms [93]. However, it is important to note that the activation of Nrf2-ARE pathway can interfere with ferroptosis-targeting therapies. Thus, the potential for unintended consequences of increasing intracellular heme levels to selectively target tumor cells warrants attention, along with evaluating the safety profiles of such approaches across diverse types of tumor cells.

## 5 The role of heme in the effects of artemisinin on other diseases

Artemisinin has garnered considerable attention owing to its multifaceted therapeutic potential, distinguished by its remarkable efficacy and minimal toxicity. It is widely employed in the treatment of malaria and exhibits promising antitumor properties in the management of human cancers. Furthermore, it has been suggested for use in addressing inflammatory diseases, as well as a range of diseases induced by viral, bacterial, fungal, and parasitic infections [19].

### 5.1 The mechanisms underlying the effects of artemisinin on non-malaria diseases

The anti-inflammatory activity of artemisinin has been widely studied in diverse models of inflammatory disease, such as allergic and suppurative inflammation [8]. Their anti-inflammatory activity is attributed to the inhibition of mitogen-activated protein kinase (MAPK), NF-κB activation, PI3K/Akt signaling cascade, and the expression of Toll-like receptor 4 (TLR4) and TLR9 [120].Kim et al. found artemisinin derived from extracts effectively inhibited the production of nitric oxide and prostaglandin E2, both of which are inflammatory mediators. Additionally, it was found to suppress the generation of pro-inflammatory cytokines, including interleukin-1, interleukin-6, and interleukin-10 [121].

Researchers have postulated numerous potential mechanisms of action for artemisinin, yet only a select few have been effectively investigated in vivo, primarily concerning the induction of oxidative stress and the exertion of anti-inflammatory effects [19]. Oxidative stress is regarded as one of the primary contributors to neurodegenerative disorders, including Alzheimer's disease [122]. Research has demonstrated that artemisinin and its derivatives exhibit neuroprotective properties, while the aberrant accumulation of amyloid beta peptide is identified as a principal factor in the pathogenesis of Alzheimer's disease [123]. Artemisinin diminished oxidative stress induced by Aβ25-35 in PC12 cells and mitigated toxicity mediated by the production of ROS. Research has shown that artemisinin can reduce Aβ-induced damage to PC12 cells through multiple mechanisms, such as inhibiting LDH release, suppressing intracellular ROS production, and regulating the Δψm and caspase 3/7 dependent pathways [124,125]. Additionally, artemisinin inhibits the intrinsic mitochondrial apoptosis mediated by Aβ by activating the ERK1/2 kinase signaling pathway [126]. The ERK1/2 pathway is a key signaling component that induces cellular antioxidant mechanisms [127]. By promoting ERK1/2 phosphorylation, artemisinin confers neuroprotective effects, underscoring the potential role of its antioxidant properties in safeguarding neuronal cells from death [126]. Artemisinin can also reduce the activity of SERCA, thereby increasing the intracellular concentration of calcium in cells [128], which, in turn, foster neurite growth through the ERK signaling pathway [129].

Artemisinin has also been employed in the treatment of autoimmune disorders, including systemic lupus erythematosus and rheumatoid arthritis. Autoimmune diseases are characterized by the activation and survival of autoreactive T cells, due to the persistent presence of autoantigens. The proliferation of these autoreactive T cells plays a crucial role in various autoimmune conditions [130]. Artemether has been shown to inhibit T cell division, typically exerting control during the G0/G1 phase of the cell cycle. This effect is related to the decrease of cell cycle regulatory proteins in the G1-phase and the increase of the CDK inhibitor p27^kip^ mediated by artemether. These findings suggest that artemether affects T cell activation and inhibits T cell cycle progression [131]. In addition, macrophages serve as pivotal effector cells downstream of T cell activation in many autoimmune diseases. Artemisinin has demonstrated the ability to inhibit the production of TNF α by peritoneal macrophages stimulated with LPS through the inhibition of NF-κB nuclear translocation [132,133]. By targeting various components of the immune system, artemisinin elicits synergistic immunosuppressive effects in inflammatory and autoimmune conditions [134].

### 5.2 The synergistic role of heme with artemisinin in antiviral efficacy

Certain studies indicated that the antiviral mechanism of artemisinin extract may result from its ability to inhibit specific viral enzymes, suppress replication of viral genetic material, and obstruct viral protein synthesis through interactions with cellular molecules [135]. Numerous investigations have revealed that artemisinin possesses the capacity to inhibit the replication of cytomegalovirus (HCMV) in both in vivo and in vitro settings, thereby demonstrating its efficacy in combating the virus [136,137]. In the presence of artemisinin, the protein levels and DNA-binding activity of virus-induced transcription factors Sp1 and NF kappaB were significantly diminished, while the cell signaling transduction kinase phosphoinositol 3-kinase was inhibited, thereby thwarting the replication of HCMV [136,138]. In the process by which artemisinin exhibits its remarkable antiviral properties, heme likewise plays an indispensable role in specific diseases. It significantly amplifies the therapeutic efficacy of artemisinin through synergistic action, thereby enhancing its disease-fighting capabilities [139]. For instance, hepatitis C virus (HCV) is an enveloped single-stranded (+) RNA virus, and research has demonstrated that both artemisinin and heme exhibit certain antiviral effects when administered independently [140]. Artemisinin inhibited HCV replication in a concentration-dependent manner, and its anti-HCV activity may be directly linked to the generation of ROS and the alkylation of HCV proteins [139,141]. Meanwhile, heme functions as an iron donor capable of suppressing HCV replicon replication by inhibiting HCV RNA-dependent RNA polymerase [142]. Their combined application could yield synergistic antiviral effects against the hepatitis C virus within a specific range, thereby enhancing the anti-HCV efficacy of artemisinin [139].

### 5.3 Heme-activated mechanisms of artemisinin against parasitic diseases

In addition to its widespread application as an antimalarial agent, artemisinin has demonstrated notable therapeutic effects in the treatment of other parasitic diseases. Given that both malaria and schistosomiasis are instigated by blood-borne parasites, some researchers theorize that the efficacy of artemisinin against schistosomiasis may stem from its ability to induce the generation of free radicals through heme [19]. *Schistosomes* have developed specific defense mechanisms to survive in the liver, which are intricately linked to the expression of antioxidant enzymes, including superoxide dismutase (SOD), glutathione reductase (GR), glutathione S-transferase (GST), and glutathione peroxidase (GPx) [143]. The combined treatment of artemisinin and heme significantly enhanced the inhibitory effects on GST, GPx, and SOD enzymes in infected mice. Notably, this inhibitory influence on these enzymes was markedly greater compared to the enzyme activity observed in the schistosomiasis recovery group receiving only artemisinin treatment [144]. Another study revealed that artemisinin and heme can exert a synergistic toxic effect on worms when administered in combination [145]. Artemisinin may interact with elevated concentrations of heme, which is derived from hemoglobin digestion by parasites, leading to the generation of iron-dependent free radicals that are detrimental to the parasites [146]. Research has demonstrated that artemisinin also imparts a noteworthy therapeutic effect in the treatment of leishmaniasis via heme [147]. Certain research findings suggest that heme serves as a crucial activator of artemisinin in the flagella of *Leishmania* parasites. Studies have revealed that heme can activate artemisinin either through heme oxygenase-like enzyme activity or through chemical interactions between heme and artemisinin [148]. This corroborates the hypotheses concerning the mechanism of action of artemisinin and its potential as an efficacious treatment for eradicating *Leishmania* parasites [[149], [150], [151]].

### 5.4 Summary

Artemisinin, celebrated for its remarkable efficacy and low toxicity, has garnered significant attention for its multifaceted therapeutic potential (see Table 1). In the realm of antiviral activity, artemisinin and heme exhibit a synergistic effect against the hepatitis C virus when utilized in tandem, while simultaneously demonstrating the capacity to inhibit the replication of cytomegalovirus. Furthermore, artemisinin has exhibited noteworthy therapeutic benefits in the management of other parasitic diseases, such as schistosomiasis and leishmaniasis, with heme also playing a pivotal role in these treatment modalities. Consequently, artemisinin presents expansive prospects for application in the treatment of various ailments, promising to inspire innovative ideas and methodologies for the development of novel, efficient, and low-toxicity therapeutic agents.

**Table 1:** The mechanisms underlying the effects of artemisinin on non-malaria diseases.

Disease/Condition	Mechanism	Reference
Inflammation	Inhibit the production of inflammatory mediators and pro-inflammatory cytokines	(118)
Alzheimer's disease	Reduce oxidative stress to promote ERK1/2 phosphorylation and activate signaling pathways to protect cells	(120) (123)
Systemic lupus erythematosus and rheumatoid arthritis	Regulating the function of immune cells and the production of antibodies	(128)
HCMV	Reduce transcription factor levels and DNA binding activity, while inhibiting phosphoinositol 3-kinase to block HCMV replication.	(133) (134) (135)
HCV	Inhibition of HCV replication by generating ROS and alkylating HCV proteins, while heme inhibits RNA polymerase to enhance its efficacy	(136) (137) (138) (139)
Schistosomiasis	Inhibit the activity of antioxidant enzymes	(140) (141)
Worms	Generate iron dependent free radicals that are harmful to parasites and have toxic effects	(142) (143)
Leishmaniasis	Activate artemisinin through heme oxygenase like enzyme activity or chemical interactions	(144) (145) (146) (147) (148)

## 6 Conclusions

Artemisinins is a class of potent bioactive compounds endowed with antimalarial, antifungal, antitumor, antiviral, and anti-inflammatory properties, while heme plays an indispensable role in the manifestation of artemisinin's pharmacological effects [24]. Many researchers mainly focused on the multiple pharmacological attributes of artemisinin or antimalarial mechanisms of artemisinin. However, these reviews do not mainly focus on the key developments in the role of heme for artemisinin multiple activities. Further, what readers need most is new insights as to how heme as activator and target for artemisinin action should be taken. Artemisinin exerts its therapeutic effect by alkylating heme and forming complexes with heme within the malaria pathogen [24,42]. In the context of anti-fungal activity, heme has been recognized for its role in mediating the activation of artemisinin [68] acting as an essential component of mitochondrial cytochrome *c* [71]. In terms of anti-cancer activity, the concentration of heme is markedly elevated in comparison to that found in normal cells [87]. Free heme, serving as the primary activator of artemisinin [88], alkylates proteins and macromolecules within the cells, leading to the fragmentation of DNA. Simultaneously, the accumulation of ROS amplifies apoptotic signals, culminating in the demise of tumor cells [44,86]. Meanwhile, in the treatment of non-malarial diseases, heme exerts a synergistic effect on the management of Alzheimer's disease and viral infections by augmenting the mechanism of action of artemisinin [126,139]. For parasitic diseases, heme serves as a pivotal activator, facilitating the generation of free radicals detrimental to parasites through artemisinin, thereby enhancing the therapeutic efficacy [[144], [145], [146]].

At present, artemisinin and its derivatives stand as frontline antimalarial agents owing to their distinctive advantages. In addition to the chemical modifications of monomeric artemisinin aimed at producing more potent antimalarial analogs, various artemisinin dimers, trimers, and even tetramers have been discovered to demonstrate enhanced antimalarial, anticancer, and antiviral efficacy throughout the research process. Nevertheless, the mechanisms through which these oligomers exert their effects have yet to be elucidated [8]. Future investigations into artemisinin will seek to uncover its therapeutic potential in disease models extending beyond malaria, encompassing infectious diseases, cancer, and immune disorders. With dedicated efforts directed toward the development of innovative synthetic artemisinin analogs and a deeper understanding of the clinical pharmacology of artemisinin, it is likely that artemisinin-based therapeutics will emerge as significant interventions for the prevention and treatment of a diverse array of human ailments beyond malaria.

## Declaration of generative AI in scientific writing

The authors declare that they didn't use generative AI in scientific writing.

## CRediT authorship contribution statement

**Pan Zhu:** Conceptualization, Funding acquisition, Supervision, Writing – original draft, Writing – review & editing. **Xinyi Sun:** Writing – review & editing. **Yuting Fang:** Writing – review & editing. **Yufei Li:** Writing – review & editing. **Liang Guo:** Conceptualization, Funding acquisition, Supervision, Writing – original draft, Writing – review & editing.

## Declaration of competing interest

The authors declare that they have no known competing financial interests or personal relationships that could have appeared to influence the work reported in this paper.

## Data Availability

No data was used for the research described in the article.
